# Recent Advances in the Clinical Translation of Silicon Fluoride Acceptor (SiFA) ^18^F-Radiopharmaceuticals

**DOI:** 10.3390/ph14070701

**Published:** 2021-07-20

**Authors:** Lexi Gower-Fry, Travis Kronemann, Andreas Dorian, Yinglan Pu, Carolin Jaworski, Carmen Wängler, Peter Bartenstein, Leonie Beyer, Simon Lindner, Klaus Jurkschat, Björn Wängler, Justin J. Bailey, Ralf Schirrmacher

**Affiliations:** 1 Department of Oncology, Division of Oncological Imaging, University of Alberta, Edmonton, AB T6G 1Z2, Canada; gowerfry@ualberta.ca (L.G.-F.); kroneman@ualberta.ca (T.K.); adorian@ualberta.ca (A.D.); yinglan2@ualberta.ca (Y.P.); cjaworsk@ualberta.ca (C.J.); jjbailey@ualberta.ca (J.J.B.); 2 Biomedical Chemistry, Department of Clinical Radiology and Nuclear Medicine, Medical Faculty Mannheim of Heidelberg University, Theodor-Kutzer-Ufer 1-3, 68167 Mannheim, Germany; Carmen.Waengler@medma.uni-heidelberg.de; 3 Department of Nuclear Medicine, University Hospital, LMU Munich, Marchioninistraße 15, 81377 Munich, Germany; Peter.Bartenstein@med.uni-muenchen.de (P.B.); Leonie.Beyer@med.uni-muenchen.de (L.B.); Simon.Lindner@med.uni-muenchen.de (S.L.); 4 Fakultät für Chemie und Chemische Biologie, Technische Universität Dortmund, 44227 Dortmund, Germany; klaus.jurkschat@tu-dortmund.de; 5 Molecular Imaging and Radiochemistry, Department of Clinical Radiology and Nuclear Medicine, Medical Faculty Mannheim of Heidelberg University, Theodor-Kutzer-Ufer 1-3, 68167 Mannheim, Germany; Bjoern.Waengler@medma.uni-heidelberg.de

**Keywords:** fluorine-18, positron emission tomography (PET), silicon fluoride acceptor (SiFA), radiochemistry

## Abstract

The incorporation of silicon fluoride acceptor (SiFA) moieties into a variety of molecules, such as peptides, proteins and biologically relevant small molecules, has improved the generation of ^18^F-radiopharmaceuticals for medical imaging. The efficient isotopic exchange radiofluorination process, in combination with the enhanced [^18^F]SiFA in vivo stability, make it a suitable strategy for fluorine-18 incorporation. This review will highlight the clinical applicability of [^18^F]SiFA-labeled compounds and discuss the significant radiotracers currently in clinical use.

## 1. Introduction

Positron emission tomography (PET) is an important modality in medical imaging. It works via the introduction of applicable positron (β^+^)-emitting radionuclides into suitable chemical vectors. The PET scanner surrounding a patient detects the two characteristic gamma (γ) rays that are produced upon the annihilation of an emitted β^+^ with a surrounding electron [1,2,3,4]. These can then be used to generate 3D images depicting the localization of the radio-pharmaceutical, which consequently assists in the detection of tumors and metastases, thus facilitating clinical diagnosis, pre-therapeutic dosimetry and treatment assessments [3,5,6].

Fluorine-18 (^18^F) is a prevalent β^+^ emitter that is used in PET imaging. It features attractive intrinsic properties, with its low mean β^+^ energy (250 keV), and half-life (t_1/2_) of 110 min, which allow for superior image resolution and prolonged imaging acquisition [4,7,8]. The t_1/2_ is ideal for use in radiotracers as it is long enough for the synthesis, transportation to a clinical center from an off-site facility and in vivo distribution, but short enough to avoid unnecessary radioactivity exposure to the patient [9]. ^18^F radiotracers play an important role in cancer detection, neuroimaging and metabolic pathway visualization [6,8]. The most well-known is ^18^F-FDG (fluorodeoxyglucose), a staple in clinical oncology, used for whole-body tumor visualization [6,10,11]. As a glucose analogue, it has a higher uptake by cancerous tissues, in which the cells have an intrinsically high metabolic rate linked to the high degree of cell division. Specifically, ^18^F-FDG is recognized by glucose transporters, which are typically upregulated by malignant tumor cells [5,6,12]. Following internalization, ^18^F-FDG is phosphorylated by hexokinase, yet it is blocked from entering glycolysis due to the 2’-fluorine substitution, which results in cellular radiotracer accumulation [5,6,12]. Since all cells have glucose transporters, this method is not very specific and is used primarily for cancer staging, which evaluates the extent of disease progression [5,10,13]. Importantly, the incorporation of ^18^F into new compounds with high target specificity allows for high-precision medical imaging and is a rapidly growing area of research. An ^18^F-labeling moiety is generally combined with a biological targeting vector, such as a small molecule, peptide or protein, which delivers the β^+^-emitting radionuclide selectively to the target tissue by means of a specific pathological marker on the cell that differentiates it from healthy cells [12,14]. This is a critical aspect for image quality, as it limits background noise caused by non-specific cellular uptake [12].

Since the successful incorporation of ^18^F into hypofluorite compounds by Cady et al. in 1948, there have been many improvements to ^18^F-labeling methodologies [11,15]. However, it was not until more recently that new advances have been made that significantly improved the efficiency and applicability of the labeling process, resulting in a surge of new PET radiotracer development. Among these numerous upgrades, the establishment of non-canonical, late-stage fluorination labeling methods involving Si-^18^F, B-^18^F and Al-^18^F bond formation have been instrumental in advancing the field [16,17,18,19,20,21,22,23,24]. In particular, these methods have been used for the kit-like production of complex ^18^F-compounds without any tedious purification processes [4]. As such, many radiotracers implementing these strategies are currently in clinical use.

This review article focuses on silicon fluoride acceptor (SiFA) radiochemistry, which is a method of ^18^F-labeling that exploits isotopic exchange (IE) such that labeling reactions take place quickly under mild conditions with undemanding techniques, resulting in high molar activity (A_m_) and radiochemical yields (RCY) [25,26,27,28,29]. As such, the use of SiFAs in peptide-, protein- and small-molecule-containing ^18^F-radiopharmaceuticals and their respective clinical translations will be further described. This includes the recently developed radiohybrid ligands, which link ^18^F PET imaging with radioligand therapy for the first time [30,31,32,33]. The emphasis of this review is on the development of [^18^F]SiTATE (TATE = Tyr^3^-octreotate), a novel SiFA ^18^F-labeled radiopharmaceutical that provides exemplary PET images of neuroendocrine tumors (NETs) in patients [29,34,35,36,37,38,39,40]. This will be the first review of its kind to highlight [^18^F]SiTATE clinical applications, including recent clinical studies, automation advances and comparisons against clinically available [^68^Ga]Ga-DOTA-TOC and [^68^Ga]Ga-DOTATATE (Netspot^®^, approved radiopharmaceutical).

## 2. Background

### 2.1. Non-Canonical ^18^F-Labeling Methodologies

Previously, ^18^F incorporation into radiotracers required nucleophilic or electrophilic substitution chemistry, which involves the formation of C-^18^F bonds [14]. Some electrophilic approaches, such as the carrier-added production of [^18^F]F_2_, are known to have some major drawbacks, such as low A_m_ and RCY [14,41]. However, methods are being produced to circumvent this issue, such as the production of ^18^F-Selectfluor by Gouverneur et al. [14,41,42]. Other drawbacks include complicated production procedures and the generation of radioactive byproducts that require a more time-intensive and costly purification process, such as high-performance liquid chromatography (HPLC) [14]. In order for ^18^F-radiotracer development to become more clinically applicable, new methodologies were required for them to be comparable to the kit-like preparation of single photon emission computed tomography (SPECT) radiotracers (e.g., technetium-99m (^99m^Tc) radiopharmaceuticals). Moreover, labeling procedures should ideally be one-step procedures, with fast reaction times and mild conditions, requiring little purification, and they should be reliable, specific and yield high A_m_ and RCY. This was accomplished through the emergence of Si-^18^F, B-^18^F and Al-^18^F labeling strategies [16,17,18,19,20,21,22,23,24]. However, all of these strategies have only showed applicability towards labeling larger compounds such as peptides and proteins. The building blocks (BB) needed for the introduction of ^18^F are rather large (with boron BB being the smallest), lending themselves only to the derivatization of compounds with higher molecular weight, while preserving the vector’s biological integrity.

B-^18^F labeling methods offer an advantage, as boron-fluorine bonds are known to be very strong (~580 kJ/mol) [9,17]. The Perrin group derived a method to produce ^18^F-organotrifluoroborates using boronic ester moieties, which provided a proof-of-principle that allowed for the development of many precursors using B-^18^F labeling [17,18]. Alternatively, McBride et al. developed a strategy that exploits a radiometal-chelator concept using Al^18^F and NOTA (p-SCN-Bn-1,4,7-triazacyclononane-1,4,7-triacetic acid) [20]. This concept utilizes the principle that ^18^F forms a stable complex with Al, which can then be linked to a targeting vector through the Al-bound NOTA chelator. Moreover, Si-^18^F chemistry was developed by Ametamey et al. simultaneously with the SiFA BB methodology by the Schirrmacher, Wängler and Jurkschat groups [8,28,43,44].

### 2.2. ^18^F-SiFA Development and Its Limitations

Although radiochemistry implementing the use of silicon-bound ^18^F dates back to 1958, its clinical translation has been only recently established [45]. Even though the Si-F bond has a higher bond energy than C-F (~90 kJ higher), the highly polar bond is quite susceptible to hydrolysis [8]. The adverse consequences due to the degradation of the Si-F bond were observed in in vivo rat studies performed by Rosenthal et al. in 1985 [46]. The [^18^F]fluorotrimethylsilane administered was converted rapidly into a silanole, and the accumulation of free ^18^F^-^ was observed in the bones. It was therefore speculated that bulky, sterically hindered groups could be a solution to this issue as they would shield the Si-F bond, protecting it from hydrolysis [8,46]. This was confirmed by the work done by Blower et al. as well as Schirrmacher and Jurkschat, which led to the development of SiFA BB synthesis and its labeling protocol [9,44]. In vivo analysis of simple molecules containing Si-^18^F with varying bulky substituents eventually lead to the discovery that triorganofluorosilanes with one aryl group and two *tert*-butyl groups were highly stable and therefore made good candidates for an ^18^F-labeling synthon [8].

Furthermore, Schirrmacher and Jurkschat were able to prove that IE is an applicable method for the labeling procedure as it conveniently results in the exchange of a non-radioactive ^19^F atom to radioactive ^18^F in one quick step with mild conditions and without the need for complicated purification procedures [44]. One main reason why IE was considered unfeasible is due to its association with a low A_m_, resulting from the large differences in concentration between the ^19^F-precursor and ^18^F anion, which would inhibit its usefulness in a clinical context [8,14,25,26]. This is because issues arise when there are significantly more non-radioactive ^19^F-bound pharmaceuticals than the radioactive ^18^F, especially in cases where the biological targets are expressed in low concentrations [14,47]. This could lead to saturation of the targets with non-radiolabeled agents, which would be detrimental to the acquisition of adequate PET images [14,47]. Additionally, low A_m_ means that a higher quantity of pharmaceutical agents would need to be injected into a patient in order to produce a sufficient image, which could have unfavorable consequences in vivo [47]. Further, it is a general rule of thumb that only 1% of receptors should be bound by an imaging agent to negate any potential activation that could arise [48]. Therefore, the determination that the IE methodology of ^18^F-labeling can result in high A_m_ as well as high RCY was a significant finding [9,44]. An initial [^18^F]triorganofluorosilane compound ([^18^F]fluorodi-*tert*-butylphenylsilane) investigated by Jurkschat et al. produced a high RCY of 80–95% after only 15 min and A_m_ values as high as 194–230 GBq·μmol^−1^, within the range typical for clinical radiotracers (100–1000 GBq·μmol^−1^) [44]. This can be attributed to the low precursor concentration (1 μg, 4.1 nM) and the fast ^19^F-^18^F exchange rate, resulting in a highly efficient ^18^F-labeling strategy [44,49]. Additionally, the preliminary in vitro human serum stability (>90%) and in vivo stability in rats were determined to be very high, with little evidence of ^18^F accumulation in the bones, a sign of ^18^F dissociation [44,49]. Notably, the SiFA-peptide conjugate SiFA-TATE was subjected to IE to observe the effect of acidic groups on the RCY [39]. Acidic groups usually pose a potential problem as they could interact with nucleophilic ^18^F^-^ and prevent the desired labeling reaction from taking place. Especially at higher reaction temperatures, the formation of H[^18^F]F leads to a significant loss of starting radioactivity. ^18^F IE with SiFA-TATE at room temperature for 10 min resulted in a very high RCY and RCP of 95% and 98%, respectively, further reinforcing its potential as a PET radiotracer labeling method [25,39,44,49].

Unfortunately, SiFA’s high stability against hydrolysis and fast and specific labeling procedure comes with a profound limitation—its high inherent lipophilicity, stemming from the SiFA BB. This lipophilicity problem can negatively affect the ability of the radiotracer to get to target areas. Specifically, if the compound is too lipophilic it will be subjected to the first pass effect, meaning it will become trapped in the liver and metabolized in order to be excreted from the organism [8]. Thus, proper biodistribution will be limited, resulting in inhibition of the ability of the radiotracer to interact with its biological target, and consequently the generation of a satisfactory PET image. This issue was directly observed when an in vivo study of bis-*tert*-butyl SiFA showed its accumulation in the liver, thus impeding its target binding [8]. Since this discovery, many efforts have been made to overcome this limitation, which include adding charged or hydrophilic groups to the SiFA BB and the targeting vector. Importantly, Wängler et al. were able to decrease the lipophilicity of a potential radiotracer consisting of a SiFA-TATE conjugate by adding a polyethylene glycol (PEG) group, two aspartic acids and a positively-charged SiFA BB [39]. This radiotracer, currently in clinical use, has been called “[^18^F]SiTATE” (previously known as [^18^F]SiFA*lin*-TATE), and will be discussed in detail in a later section.

## 3. Synthesis

**Figure 1:**
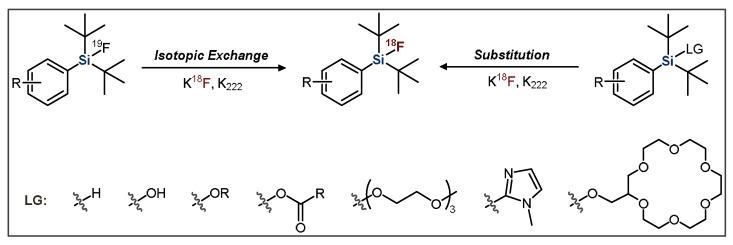
General scheme for the radiofluorination of SiFA compounds. LG = leaving group.

One of the two distinct synthetic approaches that are used in the production of Si−^18^F-labeled compounds is the leaving group (LG) approach, which uses a precursor with the silicon bonded to one of various labile moieties (Figure 1). This method was pioneered by the Ametamey group, with an initial report showing that *t*-butyldiphenylmethoxysilane could be rapidly substituted with aqueous fluoride to form [^18^F]*t*-butyldiphenylfluorosilane [43].

**Figure 2:**
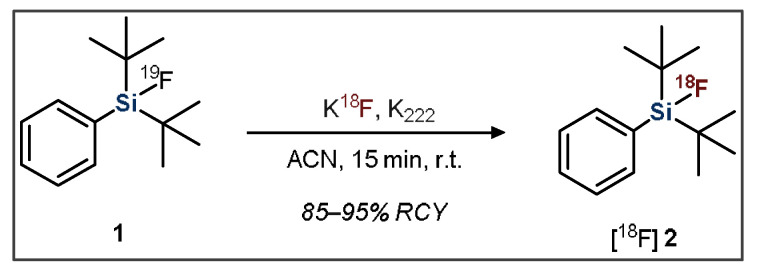
General scheme showing the radiofluorination of a dialkylfluorosilane SiFA compound.

The second approach, developed by Schirrmacher et al., is the previously introduced IE approach, which typically proceeds at room temperature, resulting in a lower amount of side-product formation (Figure 2) [44]. IE also facilitates rapid purification because the [^18^F]SiFA product is chemically identical to the ^19^F-containing starting material. This purification procedure consists of flushing the reaction mixture through a C18 solid phase cartridge (SPE), which separates the un-reacted [^18^F]fluoride from the desired product. The radiolabeled organic compound stays on the resin, whereas impurities are flushed out. Subsequently, using an elution solution, the remaining radiolabeled product is eluted from the SPE in high purity [26,27,49]. This is significant because it removes the need for more time-consuming and cumbersome purification methods such as HPLC.

**Figure 3:**
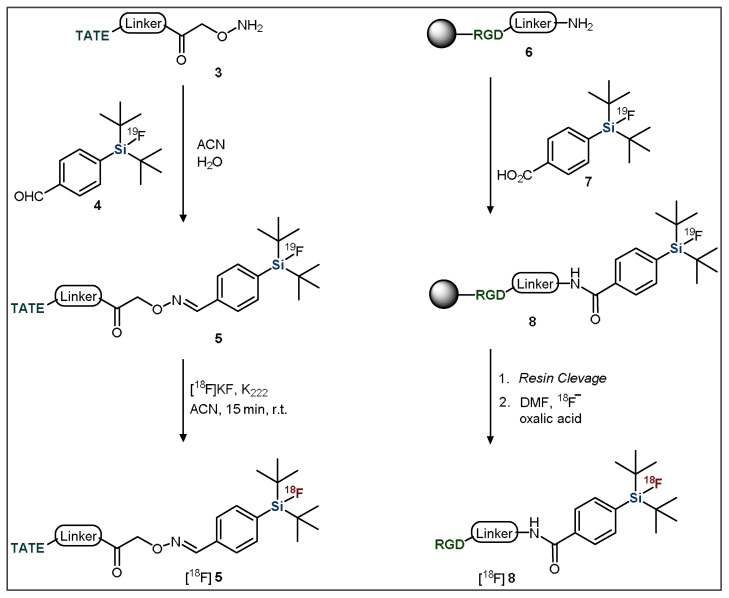
General methods for the introduction of SiFA building blocks to targeting vectors. TATE = Tyr^3^-octreotate; RGD = arginine-glycine-aspartate.

Introduction of the ^19^F-SiFA moiety into a precursor of interest is typically achieved through the coupling of a derivatized compound to a small, functionalized silyl arene [49]. For example, SiFA-TATE was produced through the reaction of an amino-oxy functionalized TATE group (**3**) with an aldehyde-derivatized SiFA moiety (**4**) (Figure 3, left) [44]. Another common strategy uses peptide bond formation to introduce the SiFA group, such as Fluciclatide (**6**) coupling with a carboxylic acid-bearing SiFA BB (**7**) using solid-phase-peptide-synthesis conditions (Figure 3, right) [50].

**Figure 4:**
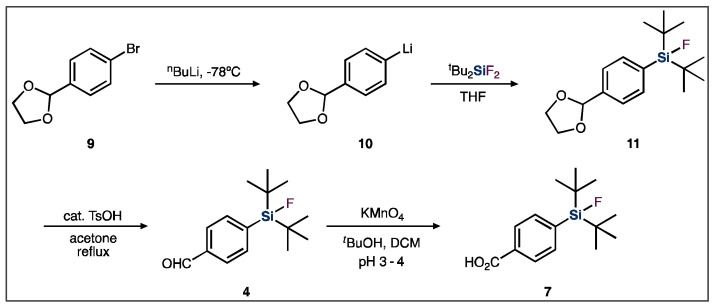
General SiFA building block synthetic scheme used for SiFA-peptide syntheses.

In order to synthesize the SiFA BB, a silyl-protected aryl bromide (**9**) is first treated with *tert*-butyl lithium, resulting in a lithium halogen-exchange reaction (Figure 4). The resulting aryl lithium compound (**10**) is then added to a di-*tert*-butyldifluorosilane, which undergoes a salt metathesis reaction. In this reaction, the carbanion acts as a strong nucleophile, resulting in the substitution of one of the silicon-bound fluoride atoms, subsequently generating the silicon-carbon bond. Finally, a deprotection step is required to couple the functionalized SiFA BB to a targeting vector of interest [8,49].

## 4. Peptides

### 4.1. SiFA Peptide Radiopharmaceuticals

The use of peptide biomolecules in nuclear medicine is continuously expanding. Many interact with pathologically significant enzymes and receptors in a highly specific manner. As such, peptide-based radiopharmaceuticals have been used for PET-based tumor visualization with a high tumor-to-background ratio, and are also utilized in peptide receptor radionuclide therapy (PRRT) as a form of cancer treatment [51,52]. Such radiopharmaceuticals have been used for imaging neuroendocrine tumors (NETs) by targeting somatostatin receptors (SSTRs), which are characteristically over-expressed on the cell surfaces of these tumor types [52]. Since the endogenous ligand somatostatin has a very short biological t_1/2_, unsuitable for clinical applications (<3 min), analogues have been produced that have better pharmacological characteristics [53,54,55]. These targeting vectors are often derivatives of the somatostatin analogue octreotide, which has a considerably high affinity for the highly expressed subtype SSTR2 and a more applicable t_1/2_ of ~72–98 min. [51,52,56,57,58]. The most commonly used octreotide derivatives are the previously introduced TATE, Tyr^3^-octreotide (TOC) and 1-NaI^3^-octreotide (NOC) [58]. The ligand–receptor interaction leads to internalization of the complex, thereby resulting in accumulation of the radionuclide in the tumor sites and permitting visualization through PET imaging [52]. Furthermore, SSTR2 expression is minimal on healthy cell types, which is notable, as it results in improved image contrast and low off-target effects [59]. Thus, these SSTR-targeting PET imaging agents have allowed for improved diagnosis and treatment monitoring of NET cancer types, as per increasingly early detection as well as extended survival duration in recent decades [60].

**Figure 5:**
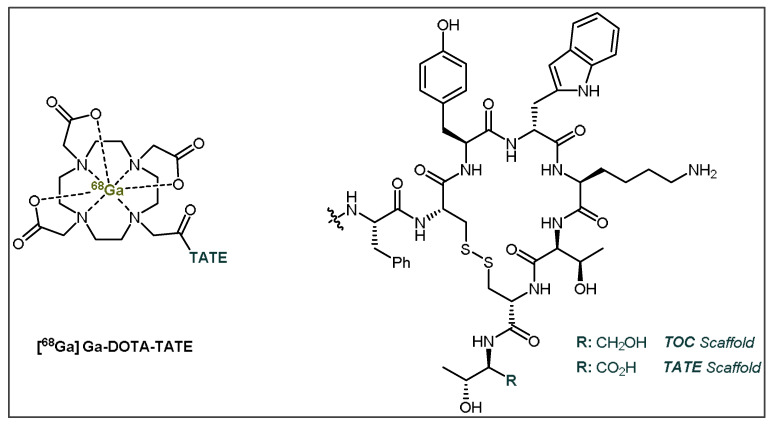
General structures of [^68^Ga]Ga-DOTA-TATE (Netspot^®^) and [^68^Ga]Ga-DOTA-TOC. TATE = Tyr^3^-octreotate; TOC = Tyr^3^-octreotide.

The current clinically used SSTR-targeting peptides for PET imaging are [^68^Ga]Ga-DOTA-TATE (Netspot^®^) and [^68^Ga]Ga-DOTA-TOC (Figure 5) [61,62]. Both tracers are labelled with gallium-68 (^68^Ga; t_1/2_ = 68 min) through the use of the well-established radiometal chelator, DOTA (1,4,7,10-tetraazacyclododecane-1,4,7,10-tetraacetic acid) [52]. Unfortunately, ^68^Ga usage inherently has some drawbacks when it comes to PET imaging [63,64]. Currently, ^68^Ga is primarily obtained from costly on-site ^68^Ge/^68^Ga-generators due to its relatively short t_1/2_. Furthermore, applicable patient numbers are constricted due to limited production amounts, although recent progress has made cyclotron production possible [64,65,66,67,68,69]. Additionally, the PET image resolution is hindered due to the high mean β^+^ energy (E_mean_ = 0.83 MeV) and concordant high mean β^+^ range (R_mean_ = 3.5 mm) of ^68^Ga [63]. ^18^F is a radionuclide that has had increased focus for SSTR-targeting peptides [37,40]. It should be noted that other novel radiometals are currently in use and are continuously being investigated due to their similar advantages over ^68^Ga, such as ^64^Cu, ^44^Sc and ^89^Zr, with ^64^Cu-DOTATOC and ^64^Cu-DOTATATE currently being in clinical use as ^68^Ga-DOTATOC and ^68^Ga-DOTATATE alternatives [70,71,72,73,74,75,76,77,78,79,80]. These radiometals also provide attractive alternatives to ^68^Ga PET imaging, although they are beyond the scope of this review.

^18^F is a cyclotron-produced radionuclide with a longer t_1/2_ than ^68^Ga, which allows for improved distribution and eases time constraints for radiopharmacists and physicians [64]. Moreover, its radiochemical t_1/2_ is a great match for use with the SSTR octreotide-derivative agonists used in NET imaging, with biological t_1/2_ of ~72–98 min [58]. This ensures that there is enough time for biodistribution of the radiotracer to target sites without a significant loss of radioactivity. Additionally, ^18^F has higher PET resolution capabilities as there is a lower mean β^+^ and range (E_mean_ = 0.25 MeV and R_mean_ = 0.6 mm, respectively) [63]. Therefore, the usage of ^18^F may be preferable over ^68^Ga for PET imaging and it has thus been introduced into various peptide-based radiopharmaceuticals for cancer imaging [40,81].

### 4.2. Utilization and Refinement of SiFA Peptides

**Figure 6:**
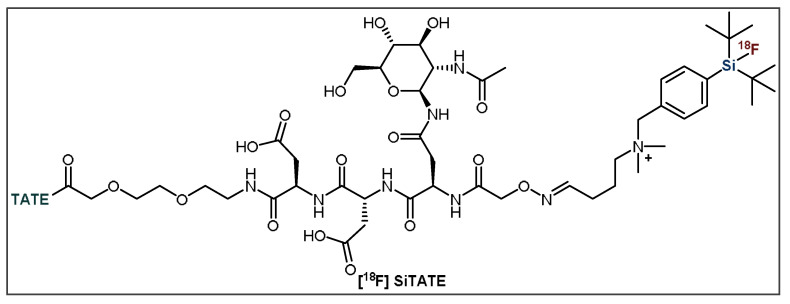
General structure of [^18^F]SiTATE. TATE = Tyr^3^-octreotate.

Despite the benefits of SiFA, the high lipophilicity of its structure is problematic for the biodistribution of radiopharmaceuticals bearing this auxiliary [8,40]. These problems have been previously exemplified through various studies that indicate high liver uptake, renal clearance and poor bioavailability, which strongly impeded the imaging capabilities of SiFA-tagged radiopharmaceuticals [26,37]. As such, efforts to make improvements in ^18^F-labeled SiFA peptides have focused upon the addition of lipophilicity-reducing constituents [37]. Due to their size and stability, peptides are often capable of tolerating these modifications without it affecting their interaction with their target [40,52]. These structural alterations may include hydrophilic chelators and carbohydrate groups, as well as the addition of polar and/or charged amino acids, which reduce the overall lipophilicity of the molecule, thereby improving biodistribution [40]. The most promising SiFA-bearing peptide radiopharmaceutical is the previously mentioned [^18^F]SiTATE (Figure 6), which bears the same peptide vector as the clinically used [^68^Ga]Ga-DOTA-TATE ((Netspot^®^), along with an Asn(AcNH-β-Glc)-PEG_1_ spacer, two aspartic acid residues and the permanently positively charged SiFA group [38,40,52].

**Figure 7:**
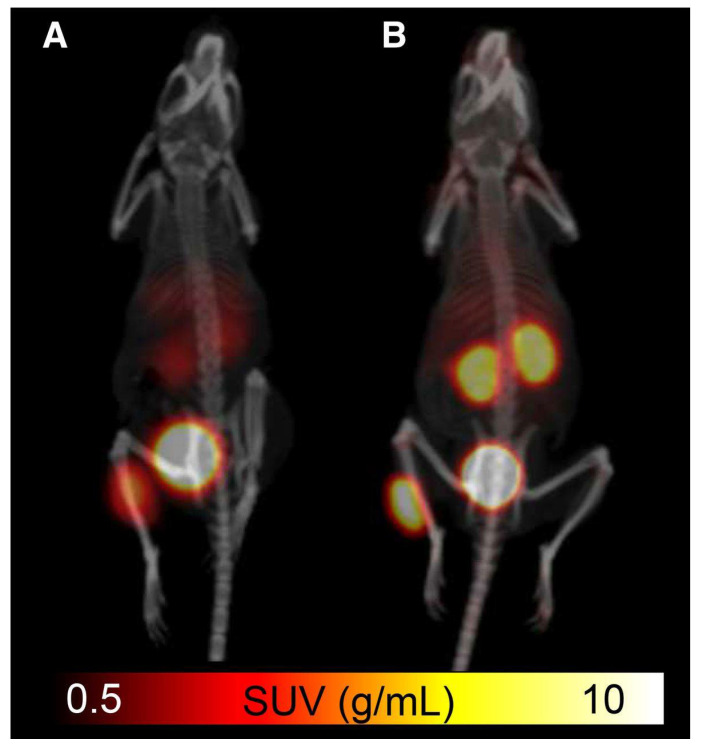
Comparison of (**A**) [^68^Ga]Ga-DOTA-TATE and (**B**) [^18^F]SiTATE in small animal PET/CT imaging with standardized uptake value (SUV) scale [37]. Reprinted with permission from Ref. [37]. Copyright 2015 SNMMI.

The refinement of [^18^F]SiTATE was accomplished through analysis of various parameters, including SSTR-binding capabilities, lipophilicity and PET image quality [37,40]. These assessments in animal models revealed that the lipophilicity-reducing modifications successfully amended previous problems among SiFA peptides, as [^18^F]SiTATE demonstrated limited liver accumulation, low background noise and higher tumor uptake, with a decrease in lipophilicity when compared to [^18^F]SiFA-TATE [40]. Additionally, a study comparing the effectiveness of [^18^F]SiTATE to [^68^Ga]Ga-DOTA-TATE in mice showed similar biodistribution profiles yet superior tumor uptake for [^18^F]SiTATE (18.51% ± 4.89% vs. 14.10% ± 4.84% ID/g, respectively) (Figure 7) [37]. These results indicate that this tracer could be an ideal candidate for future clinical studies.

### 4.3. Clinical Applicability of ^18^F-SiTATE

**Figure 8:**
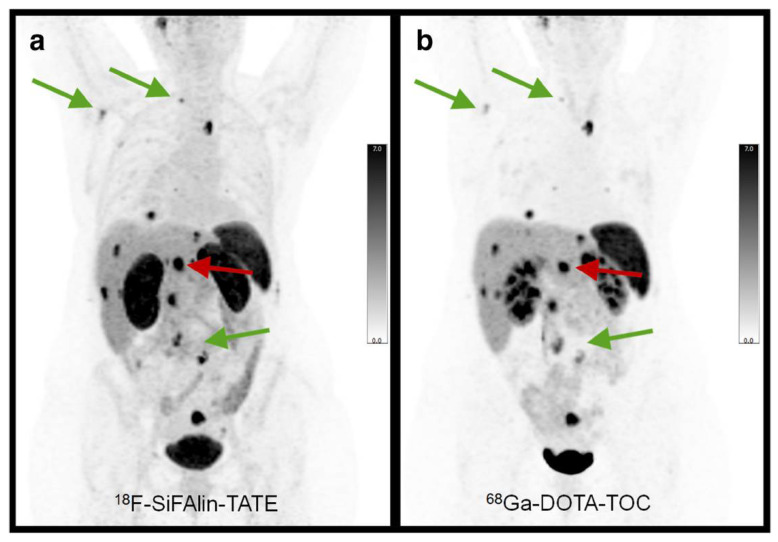
In-patient comparison of PET image quality between (**a**) [^18^F]SiTATE and (**b**) [^68^Ga]Ga-DOTA-TOC. Patient was a 39-year old female with Ileum NET. Comparison shows superior image quality with [^18^F]SiTATE, especially for small lesions. Reprinted with permission from Ref. [38]. Copyright 2019 Springer Nature.

Following its positive pre-clinical results, various assessments of [^18^F]SiTATE have indicated promising results that demonstrate its strong clinical potential in humans [34,35,38]. Specifically, these studies have compared [^18^F]SiTATE and [^68^Ga]Ga- DOTA-TOC with respect to biodistribution, tumour uptake and image quality, among other parameters that are clinically relevant for these radiotracers [34,35,38]. The first clinical assessment of [^18^F]SiTATE compared to [^68^Ga]Ga-DOTA-TOC was a study among 13 NET-positive patients that assessed biodistribution, tumor uptake and image quality using inter-observer agreement using five blinded readers [38]. This study revealed similar, yet higher uptake of [^18^F]SiTATE in healthy adrenal glands, liver and spleen, whereas it exhibited lower uptake than [^68^Ga]Ga-DOTA-TOC in the thyroid, lungs and bone, among other sites [38,82]. Despite the higher uptake of [^18^F]SiTATE into the healthy liver and spleen, this radiotracer displayed higher tumor uptake, tumor-to-spleen ratios and tumor-to liver ratios [38]. The clinical utility of this tracer was clearly exemplified in this study, as [^18^F]SiTATE imaged all 109 lesions that [^68^Ga]Ga-DOTA-TOC imaged, although with greater resolution (Figure 8). Furthermore, the blinded image readers denoted each image as being either “good” or “excellent”, with these being the second highest and highest scores, respectively.

**Figure 9:**
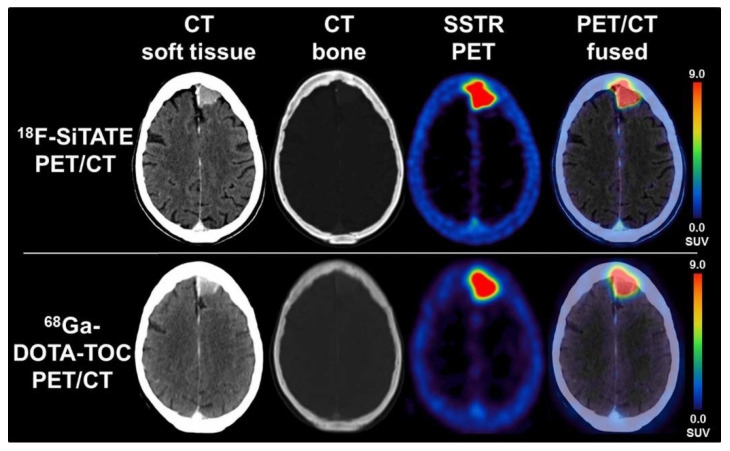
PET/CT comparison between [^18^F]SiTATE and [^68^Ga]Ga-DOTA-TOC in a patient with a falx meningioma with transosseous extension. Images show preferable resolution with [^18^F]SiTATE, therefore yielding a higher quality image. Reprinted with permission from Ref. [34]. Copyright 2021 Wolters Kluwer Health, Inc.

Subsequent [^18^F]SiTATE clinical studies have further assessed biodistribution and imaging, along with assessments of dosimetry and optimal scan times [34,35]. Dosimetry comparisons between [^18^F]SiTATE and [^68^Ga]Ga-DOTA-TOC revealed that the former delivers slightly lower required radiation doses, with activities of ~100–120 MBq being sufficient to produce a suitable image. Furthermore, these comparisons reinforced the previous findings of higher tumor-to-background ratios and image quality (Figure 9), while finding no significant differences in optimal scan-times between these tracers [35]. Collectively, these results presently indicate [^18^F]SiTATE to have a strong clinical potential for replacing [^68^Ga]Ga-DOTA-TOC or TATE (Netspot^®^) as the gold-standard SSTR-targeting peptide radiopharmaceutical. Despite many non-significant differences between these tracers, the logistical advantages of ^18^F over ^68^Ga—its higher-image resolution, cyclotron-production, and longer t_1/2_—strengthen the clinical applicability of [^18^F]SiTATE [63]. Furthermore, the recently developed automated radiosynthesis of [^18^F]SiTATE by Lindner et al. has, in conjunction with a refined SiFA methodology, reduced technical constraints, improved accessibility and enhanced its capacity to be implemented into full clinical application [29]. Importantly, clinical studies utilizing radiotracers produced via this automation method exhibited no side effects or changes in biodistribution and tumor uptake. Lastly, the relatively longer t_1/2_ of ^18^F can potentially allow for the transportation of ^18^F-SiTATE to hospitals without immediate access to a cyclotron facility, while still providing slightly preferable radiation doses compared to [^68^Ga]Ga-DOTA-TOC [35,64]. As such, [^18^F]SiTATE is an effective SiFA-bearing peptide radiopharmaceutical that has an optimistic future in the imaging of NETs, with the ultimate goal of improving patient care and prognosis.

### 4.4. Radiohybrid Ligands

In 2019, Wester et al. published their important work regarding the production of radiohybrid prostate-specific membrane antigen (rhPSMA) inhibitors, constituting a great contribution to the field of medical imaging, as well as therapeutics [32]. These radiotracers incorporate both a SiFA BB and a metal chelator. Originally, the introduction of a chelator was designed to further decrease the lipophilicity of a SiFA-based PSMA inhibitor such that it could be used to image PSMA-positive prostate cancer. The results showed that adding the chelator did significantly decrease the lipophilicity (log P −2.0 to −3.5), to an extent that had not yet been reached by even the most hydrophilic SiFA-compound previously reported, α_v_β_3_ integrin-binding RGD-peptide (log P = −2.0), and greatly lower than previously discussed [^18^F]SiTATE (log P = −1.21) [37,50]. Even more important, the chelator DOTA has shown exemplary binding characteristics towards ^68^Ga, ^111^In and ^177^Lu. The significance of this is that the same radiotracer can be used for either ^18^F or ^68^Ga PET imaging or ^111^In SPECT imaging. Therefore, clinical centers with access to either radionuclide would have the ability to produce similar images in terms of biodistribution, although these would differ in image quality and clinical significance due to the specific radionuclides’ inherent characteristics. Moreover, the therapeutic radionuclide, ^177^Lu, could be utilized due to its affinity for DOTA. Therefore, the same radiopharmaceutical could be used for both therapy and diagnostic imaging, which would be the first time ^18^F PET could be directly linked to radioligand therapy. The substantial implication of this is that pretherapeutic dosimetry would be extremely accurate since the biodistribution and pharmacological properties would be identical. The current radiotracers incorporating ^68^Ga and ^177^Lu for diagnostic imaging and radiotherapy are two chemically different compounds, which affects their dosimetry precision.

**Figure 10:**
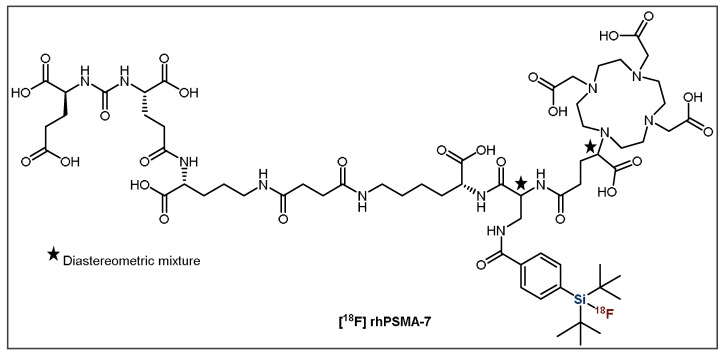
General structure of [^18^F]rhPSMA-7.

Clinical studies in 202 prostate cancer patients with one of the most promising candidates, [^18^F]rhPSMA-7 (Figure 10), showed exceptional imaging that was comparable to or even better than [^68^Ga]Ga-PSMA-11, especially in patients with low levels of PSA (prostate-specific antigen) [30,31]. Additionally, the biodistribution and tumor uptake appeared to be similar to other established PSMA ligands reported in the literature [31]. Further clinical studies in patients with high-risk prostate cancer showed that [^18^F]rhPSMA-7 has very high diagnostic accuracy, even greater than the current morphological imaging mode [33]. These results further validate its potential as an imaging agent, although further studies are required to determine its efficacy as a theranostic agent in combination with the radiometal ^177^Lu. Importantly, due to its favorable outcome, ^18^F-synthesis has been fully automated using the Munich Method, which is perfectly suited for SiFA IE [32]. This allows for the synthesis to occur with extremely high reliability (98.8%), meaning the production procedure was successful for 240/243 runs resulting in an average RCY and RCP of 50% and 99.9%, respectively [32]. This is a huge achievement and substantial step forward in the process of introducing this radiotracer into routine clinical applications.

### 4.5. GRPr Imaging

**Figure 11:**
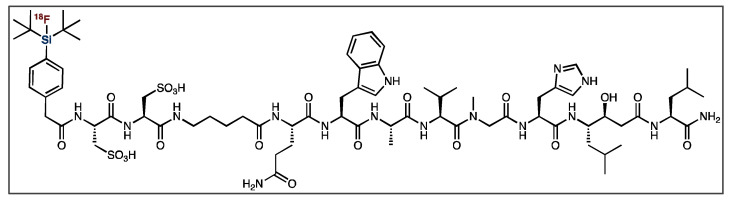
General structure of [^18^F]SiFA bombesin derivative.

Gastrin-releasing peptide receptor (GRPr) imaging is used in the diagnosis of several cancer types, including prostate cancer [83,84,85,86,87]. Bombesin shares a C-terminal amino acid sequence with GRPr’s native ligand, gastrin-releasing peptide, and is known to have a high affinity and specificity towards GRPr [83,84,88]. Therefore, bombesin derivatives have been labeled with applicable radionuclides for PET and SPECT imaging [83,84,85,86,87,88,89,90]. As with other SiFA peptides, the latest generation of analogues utilize polar auxiliaries to counterbalance the increase in lipophilicity [91]. The Ametamay group reported the synthesis of cysteic-acid-containing bombesin (Figure 11). However, despite showing improvements over the first generation of SiFA derivatives (without polar auxiliaries), it was found to have low tumor uptake, along with a poor tumor-to-blood ratio and significant hepatobiliary clearance [88].

Lindner et al. evaluated SiFA bombesin analogues with added PEG chains, along with various carbohydrate and acidic amino acid residues [50]. However, these compounds showed high liver accumulation, and were unsuccessful in imaging the target tumors. These issues might be alleviated through the introduction of the more recently discovered SiFA*lin* (SiFA BB bearing a positive charge) moiety used in SiTATE, but no such bombesin analogue has been reported to date.

### 4.6. Integrin Imaging

Angiogenesis is a fundamental process in the growth and metastasis of many cancer types. Integrin glycoproteins, in particular α_v_ß_3_ and α_v_ß_5_, are highly upregulated in vascular endothelial cells during this process and are important targets in oncological imaging [92,93]. Various probes containing the arginine-glycine-aspartic acid (RGD) amino acid motif, such as the bicyclic RGD compound fluciclatide, target these glycoproteins [94]. Lindner et al. demonstrated that the synthesis and radiofluorination of SiFA-bearing fluiclatide could yield the corresponding tracer, [^18^F]SiFA-fluciclatide, with A_m_ values up to 60 GBq·μmol^−1^ [50]. As with other SiFA tracers, polar auxiliary introduction was integral to ensure favorable pharmacokinetics, with the best containing a LysMe3-g-carboxy-d-Glu auxiliary. This tracer showed encouraging tumor uptake in tumor-bearing mice, as well as favorable biodistribution and rapid blood clearance [50]. As with the bombesin analogues, fluciclatide-based compounds have yet to be evaluated with the SiFA*lin* moiety, which may further improve their performance.

## 5. Proteins

Contrary to direct peptide [^18^F]SiFA labeling, a prosthetic group (PG) is labeled with ^18^F first and then conjugated to the protein, typically through click chemistry methods. Some studies exploiting this method involved the production of the maleimide- and thiol-bearing [^18^F]SiFA compounds [^18^F]SiFA-M and [^18^F]SiFA-SH, which were conjugated to derivatives of rat serum albumin (RSA) protein [8,95,96]. The resulting compounds were successfully produced with adequate RCYs and were therefore further subjected to small animal blood pooling studies, in which [^18^F]SiFA-M demonstrated exemplary in vivo stability [95,96]. In an effort to make the protein-[^18^F]SiFA labeling process more efficient, a SiFA-isothiocyanate derivative ([^18^F]SiFA-ITC) was generated to enable direct conjugation of the PG to lysine side chains, effectively eliminating the protein modification step [97]. Conjugation of these SiFA-PGs to various proteins resulted in favorable RCYs (~30–80%), with the percentage depending on the ratio of SiFA-PG to protein [97]. Furthermore, in small animal studies these tracers also exhibited excellent in vivo stability, shown by the very low amount of radioactivity accumulation in the bones [97]. However, one substantial drawback of this method is that these compounds have to be very carefully stored under the exclusion of water to avoid degradation of the SiFA-PG.

Additionally, a SiFA-PG was synthesized bearing an active ester group ([^18^F]SiFB). Initially, the use of an active ester-containing group was not possible as a method for bioconjugation due to the instability of that group under the basic conditions required for IE. However, Kostikov et al. were able to amend the ^18^F-labeling conditions such that the basicity was neutralized using oxalic acid [98]. This methodology allowed for the active ester SiFA compound to be labeled in high RCYs (up to 56%), while avoiding conditions that promote hydrolysis. Small animal blood pool studies were performed with [^18^F]SiFB-conjugated RSA, which showed a comparable biodistribution profile to the established protein labeling group, [^18^F]SFB, as well as in vivo stability [98].

Furthermore, Glaser et al. were able to extend the SiFA methodology to affibody labeling in 2013, generating an [^18^F]-SiFA-labeled human epidermal growth factor receptor (HER2)-targeted affibody, Z_HER2:2891_, for the imaging of HER2-positive breast cancer [99]. This affibody was coupled to a SiFA-maleimide PG through a C-terminus cysteine modification, which was then subjected to IE under aqueous conditions (water/2.5% TFA (*v*/*v*)) resulting in a RCY of ~38% [99]. This compound showed an adequate biodistribution in A431 tumor xenograft models, as well as subnanomolar binding affinity to HER2 [99]. Unfortunately, this tracer was found to undergo hydrolysis in vivo due to high ^18^F bone uptake levels and showed less than ideal tumor uptake compared to other candidates and was not carried forward into further studies. However, the ability to extend the SiFA-strategy towards large biomolecules like affibodies and exploit the positive attributes of IE in this way is noteworthy and might lead to more improved labeling protocols in the future.

## 6. Small Molecules

The significant increase in lipophilicity when the SiFA moiety is introduced into targeting vectors is even more predominant for small molecules due to the size similarity. Therefore, there are not many small molecules utilized with a SiFA BB and only four classes have been reported recently, which were described in previous reviews [8,49]. The first [^18^F]SiFA-labeled small molecules, nitroimidazole analogues of [^18^F]FMISO used for the detection of hypoxia, were reported by Bohn et al. [100,101]. The authors looked at the hydrolytic stability of many [^18^F]SiFA-FMISO alkylated analogues, yet only the *t*-Bu_2_Ph-derivative was stable enough for in vivo studies. However, further small animal studies showed that the compound precipitated in mouse lungs due to its high lipophilicity [100].

**Figure 12:**
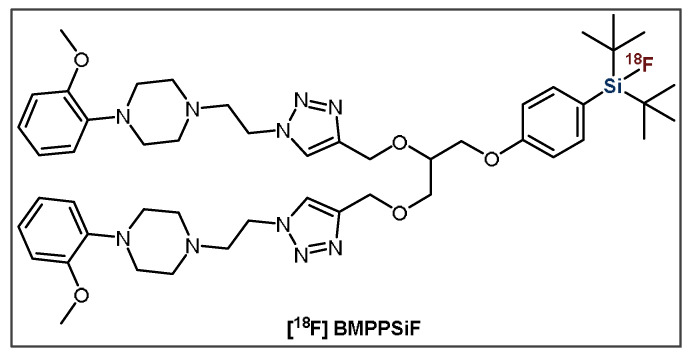
General structure of bivalent radioligand, [^18^F]BMPPSiF.

The second group of small molecules investigated by the Schulz group in 2011 were [^18^F]SiFA-labeled nucleosides and nucleotides [102]. The efficient synthesis resulted in a decent RCY of ~40%, as well as high A_m_ (>370 GBq·μmol^−1^) and RCP (~95%) values; however, higher temperatures were required. Concerns about lipophilicity have seemed to impede further studies on these compounds, yet this work does have interesting implications on the possibility of labeling related molecules such as aptamers with [^18^F]SiFA. Additionally, Wängler et al. produced *t*-Bu_2_SiF-derivatized small molecule D_2_-receptor ligands based on fallypride (FP) and desmethoxyfallypride (DMFP) in 2011 [103]. They determined that SiFA-FP, SiFA-DMFP, SiFA-M-FP and SiFA-DDMFP had reduced affinities to the D_2_-receptor in comparison to FP and DMFP; however, these were still within the nanomolar range. However, SiFA-M-FP, which consists of a short linker and thus an increased distance between the binding motif and the SiFA moiety showed relatively higher affinity to the D2 receptor compared to the other derivatives. However, its subpar chemical stability resulted in unfavorable IE results with low ^18^F-incorporation (~16.6%) and RCP (<50%), limiting its applicability [103]. More recently, Hazari et al. developed a promising PG, SiFA-dipropargyl glycerol, that was used to construct the homodimeric neuroimaging PET tracer [^18^F]BMPPSiF (Figure 12) [104]. This radiotracer comprises one SiFA moiety and two 5-HT_1A_-selective ligands that target dimeric serotonin receptors. This multimeric approach may have the potential to decrease the lipophilicity of SiFA-containing small molecules [28]. The bivalent SiFA-derivatized compound exhibited an enhanced affinity for serotonin receptors, as well as high uptake in the 5-HT_1A_-receptor-rich regions of the brain—the hippocampus, cortex and hypothalamus—in 5-HT/D2 depletion rat models [104].

## 7. Conclusions

Since initial studies in 2006 showed its improved in vivo stability against hydrolysis, the SiFA principle for the production of radiofluorinated radiopharmaceuticals has gained much interest, resulting in its incorporation into many potential radiotracers, some of which having made it into clinical applications. The IE approach offers advantages including mild reaction conditions; limited side-product generation; fast reaction times; high RCY; high molar activity and selectivity; and a quick, undemanding purification process. Furthermore, ^18^F has some preferable characteristics over ^68^Ga, such as a longer half-life and lower mean positron energy, allowing for the production of PET images with higher resolution and longer scan times. This can be instrumental in the detection of smaller lesions and metastases. Importantly, recent clinical studies have revealed comparable biodistribution and tumor uptake levels between [^18^F]SiTATE and [^68^Ga]Ga-DOTA-TOC in NET-positive patients. This demonstrates the potential of [^18^F]SiFA-conjugated peptides in terms of their full implementation into clinical PET imaging applications. Additionally, the generation of PSMA radiohybrid (rhPSMA) ligands containing a SiFA moiety and a radiometal chelator gave rise to the first potential radiotracers utilizing an ^18^F-^177^Lu theranostic pair equivalent. This is a substantial development as it greatly improves the ability to perform accurate dosimetry and pre-therapeutic assessments, further increasing its clinical applicability. Specifically, [^18^F]rhPSMA-7 was able to detect high-risk primary prostate cancer in patients with low antigen levels, making it a promising radiopharmaceutical, and it is currently in stage III clinical trials.

## Data Availability

Data sharing is not applicable to this article.
